# Procalcitonin Assay Has No Role in the Routine Assessment of Severe Trauma Patients at Admission to the Emergency Department

**DOI:** 10.7759/cureus.16228

**Published:** 2021-07-07

**Authors:** Vijaya S Lakshmi, Anusha Cherian, Prashant Adole

**Affiliations:** 1 Emergency Medicine, Jawaharlal Institute of Postgraduate Medical Education and Research, Pondicherry, IND; 2 Anaesthesiology and Critical Care, Jawaharlal Institute of Postgraduate Medical Education and Research, Pondicherry, IND; 3 Biochemistry, Jawaharlal Institute of Postgraduate Medical Education and Research, Pondicherry, IND

**Keywords:** trauma, triage, head injury, mortality, sepsis, procalcitonin, injury severity score

## Abstract

Introduction

A key challenge in emergency departments (ED) is the early recognition of sepsis or the potential for sepsis in patients. Appropriate and accurate ED triage will ensure improved case management. This study analysed the association between ED findings at admission and outcomes in patients presenting with severe trauma.

Methods

This was a prospective study conducted at a tertiary level ED and included severely injured adult patients who presented to the ED within 24 hours of injury. Data collected included clinical findings and imaging reports at initial assessment, serum procalcitonin (PCT), length of ICU and hospital stay, the incidence of bloodstream and other infections, and patient outcome as discharge from care or death. Multiple logistic regression was used to assess the association between outcome variables and independent variables.

Results

A total of 155 patients were included in the study. Head and neck (61.9%), extremity (58%), and chest (45%) were more commonly injured. Injury Severity Score (ISS) >25, Glasgow Coma Scale (GCS) score <8, head and neck injuries, and extremity injuries were found to be significantly associated with mortality. Bloodstream infections were more common in the presence of lung contusions, abdominal injury, operative management, and blood transfusions. PCT levels at admission did not have a significant predictive value for mortality, bloodstream infections, other infectious complications, or length of ICU stay.

Conclusions

Head injuries were the most common cause of mortality in our study. In addition to the anatomical region involved, ISS and GCS have a significant association with mortality. PCT levels at ED admission do not have any prognostic value and need not be routinely analysed.

## Introduction

Road traffic accidents (RTAs) contribute significantly to preventable death and illness globally. According to the WHO Global Status Report on Road Safety 2018, 1.35 million people lose their lives every year, and 20 to 50 million people get injured from RTAs. RTAs rank among the first five causes of morbidity and mortality in Southeast Asian countries. In India, an accident occurs every one minute, and a death every 3.7 minutes, amounting to more than 3.7% loss of national GDP. There has been a marked increase in the social and economic burden due to RTA in recent years [1,2].

Polytrauma patients require prolonged hospital care and are especially prone to inflammatory response syndrome and infectious complications. Despite recent medical advances, morbidity and mortality in severe trauma patients due to sepsis continue to remain high. A key challenge in emergency departments (ED) is the early recognition of the potential for or manifestation of sepsis.

Appropriate and accurate ED triage will ensure improved case management [3]. The Injury Severity Score (ISS) is a reliable prognostic tool for trauma and aids in the triaging of injured patients in large trauma centres. As an alternative, recognising the criticality of specific organ system involvement may be a more intuitive means of triaging. Recent studies have focused on the role of biomarkers such as procalcitonin (PCT) in predicting the need for higher acuity care [4,5].

This study analysed the association between ED findings at admission and outcomes for patients presenting with severe trauma, with specific reference to the role of PCT assay at admission.

## Materials and methods

This was a prospective study conducted at a tertiary level ED from July 2017 to June 2018. This study included trauma victims above 18 years of age who presented to the ED within 24 hours of trauma and had an ISS of >15. The Institutional Review Board approved this study, and all patients or their carers gave written informed consent to participate in the study.

The type and severity of trauma were classified according to the ISS. The data collected included clinical findings and imaging reports at initial assessment, length of ICU and hospital stay, the incidence of bloodstream and other infections, and the final patient outcome as discharge from care or death.

Blood samples obtained at admission were also assayed for serum PCT as a potential biomarker for severe traumatic injury. PCT assay was performed by using the sandwich enzyme-linked immune-sorbent assay technology. This has a functional assay sensitivity of <10 pg/mL [range: 31.2-2000 pg/mL; intra-assay coefficient of variation (CV) <8%, and inter-assay CV <10%].

The sample size was calculated by using nMaster software version 2.0, based on the sensitivity of PCT level of 91% from a previous study, with absolute precision of 10%, confidence interval of 95%, and prevalence of complications among trauma patients of 20%. The estimated sample size was 155 [5].

Data were collected and recorded in a spreadsheet and analysed using IBM PASW statistics 19.0 (SPSS version 19.0; IBM, Armonk, NY). Categorical variables like clinical and demographic characteristics were expressed as means with standard deviations (SD) or medians with interquartile ranges (IQR). The comparison of clinical outcomes (death or discharge) with various clinical and demographic characteristics was performed by using the Chi-square or Fisher’s exact test. The comparison of outcome variables with PCT levels was carried out using the Chi-square test. The receiver operating characteristic (ROC) curve was used to assess the sensitivity and specificity of PCT levels in identifying complications. Multiple logistic regression was used to assess the association between outcome and independent variables. The level of significance was set at 5% and a p-value <0.05 was considered to be statistically significant.

## Results

A total of 155 patients were included in the study. The cause of trauma was RTA in 76% of patients, with falls and assaults accounting for most of the remainder. Head and neck (61.9%), extremity (58%), and chest (45%) were more commonly injured. The median ISS was 25, and the median Glasgow Coma Scale (GCS) score was 13 (Table 1).

**Table 1:** Clinical features ISS: Injury Severity Score; GCS: Glasgow Coma Scale; AIS: Abbreviated Injury Score; SD: standard deviation; IQR: interquartile range

Variables	Value
Age, years, mean ± SD	40.80 ± 14.34
Serum procalcitonin, ng/dl, mean ± SD	1.33 ± 1.63
GCS score, median (IQR)	13 (6–15)
ISS, median (IQR)	25 (20–34)
Length of ICU stay, days, mean ± SD	3.86 ± 3.95
Length of hospital stay, days, mean ± SD	11.83 ± 14.43
AIS body region, n (%)	
Head and neck	96 (61.9)
Face	31 (20)
Chest	70 (45)
Abdomen	36 (23.2)
Extremity	90 (58)
External	34 (21.9)

A total of 41 (26.5%) patients died in the course of their treatment. The most common cause of death was head injury (80.5%) followed by sepsis (12.2%). On analysing factors contributing to mortality in these patients, ISS >25, GCS <8, head and neck injuries, and extremity injuries were found to be significantly associated with mortality (Table 2).

**Table 2:** Factors predicting mortality ISS: Injury Severity Score; GCS: Glasgow Coma Scale; AIS: Abbreviated Injury Score

Variables	Outcome: death	P-value
	N (%)	
ISS >25	39 (37)	<0.001
GCS ≤8	28 (56)	<0.001
AIS regions		
Head and neck	39 (41)	<0.001
Chest	15 (21)	0.198
Extremity	18 (20)	0.03
Abdominal injury	7 (19)	0.277

The strengths of association between the various factors and mortality are presented in Table 3.

**Table 3:** Strength of association: factors predicting mortality ISS: Injury Severity Score; CI: confidence interval

Variables	Unadjusted prevalence risk (95% CI)	Adjusted prevalence risk (95% CI)
ISS >25	9.29 (2.34–36.92)	5.13 (1.50–17.51)
Head and neck injury	11.9 (3–47)	-
Subdural hematoma	3.03 (1.86–4.95)	1.65 (1.03–2.64)
Subarachnoid haemorrhage	3.67 (2.34–5.72)	1.98 (1.108–3.57)
Contusion	2.25 (1.36–3.74)	1.81 (1.13–2.88)
Cerebral infarction	3.62 (2.38–5.50)	1.48 (0.76–2.87)
Skull fracture	2.56 (1.54–4.25)	1.14 (0.642–2.04)
Bloodstream infections	2.04 (1.08–3.85)	16.15 (3.46–75.2)
Cervical spine injury	2.01 (1.01–3.98)	3.15 (1.46–6.79)

Bloodstream infections were more commonly associated with lung contusions, abdominal injury, operative management, and blood transfusions. Patients with bloodstream infections tended to have a prolonged length of ICU and hospital stay. The other factor that was associated with prolonged ICU stay was hypotension on arrival.

PCT levels at admission did not have a significant predictive value for mortality, bloodstream infections, other infectious complications, or length of ICU stay (Table 4). The mean PCT level was 1.3 ng/dL. Overall, PCT levels were <0.5 ng/mL in 18 (11.6%) patients, 0.5-2 ng/mL in 111 (71.6%), and >2 ng/mL in 26 (16.8%). Using ROC curve analysis, PCT level of 0.9 ng/mL was found to have the best sensitivity (54%) and specificity (52%) for mortality, with an area under the curve (AUC) of 0.517.

**Table 4:** Potential role of serum procalcitonin as a prognostic factor ICU: intensive care unit

Outcome variable	Serum procalcitonin (ng/mL)		P-value
	>0.9	<0.9	
Death	22	19	0.552
Bloodstream infection	5	7	0.563
Other infections	23	16	0.18
Length of ICU stay >10 days	8	11	0.481

## Discussion

The present study was an observational study conducted in the ED of a tertiary care hospital to analyse admission findings that may aid in prognosticating patients with severe trauma. The study included a total of 155 patients with severe trauma. ISS >25, GCS <8, and head and neck injuries had a significant association with mortality. Sepsis, which was the second most common cause of mortality following head trauma, was associated with lung contusions, abdominal injury, operative management, and blood transfusions. Admission PCT levels did not have any significant impact on mortality, infectious complications, or length of ICU stay.

The ISS is universally accepted as a reliable predictor of mortality. Theoretically, the probability of survival decreases with increasing ISS scores. Our findings corroborate existing literature regarding the reliability of ISS in predicting outcomes and its role in ensuring better triage and resource allocation in the ED [6].

Several studies have established head injury as the most common injury encountered in major trauma centres. Also, head and spine injury patients have the highest unadjusted hazard ratio for mortality. Head injury mortality is known to be much higher in resource-poor settings. This finding highlights the demand for specialised care for patients with head injuries, in addition to timely and appropriate intervention [7].

Blood transfusions appear to have a dose-dependent relationship with infections, even after controlling for all other variables. According to the literature, each additional unit of blood transfusion increases the risk of infection by about 7% [8]. This underlines the importance of prompt haemorrhage control as a means of limiting excessive blood transfusions.

Our findings suggest that patients with lung or abdominal injury have a higher predisposition to infections. In lung injury, second insults may contribute to acute lung injury, usually owing to sepsis. The relationship between acute lung injury and sepsis is yet to be clearly established and each has the potential to contribute to the other [9]. The gut has an established reputation as the motor that drives and perpetuates multi-organ dysfunction. Understandably, abdominal injury has a higher association with bloodstream infections. The gut microbiome, intestinal hyperpermeability, and the intestinal microenvironment are likely contributory to the higher incidence of sepsis. Once again, from the ED standpoint, recognition of the presence of abdominal and chest injury should prompt a quick and decisive ED response.

PCT is an important biomarker for sepsis as it is detectable in the serum within a few hours. Its role as a rapid diagnostic test for sepsis was established in a meta-analysis [10]. Several studies have demonstrated an association between PCT levels and poor prognosis in patients with severe trauma [3,11,12]. This is supported by the rationale that serious traumatic injuries result in exaggerated systemic inflammation, which is likely to be identified by elevated PCT levels. The contrary view is that PCT levels are only mildly elevated in non-infectious inflammatory responses [13,14]. Our findings appear to support the contrary view as we failed to establish any association between admission PCT levels and the outcome variables. In our study, head trauma was the predominant injury and the most common cause of death. PCT, which is an indicator of sepsis, may have low utility in patients with fatal head trauma.

A major limitation of our study was that PCT was measured only at admission. Studies that have established the role of PCT in the management of critically ill patients have relied on serial measurements. The objective of this study was to evaluate admission PCT levels alone. Hence, only admission samples were assayed for PCT. The timing and frequency of sample collection for PCT may also be a factor that determines its relevance.

## Conclusions

Severe trauma is associated with significant mortality and morbidity. Admission findings that may indicate a higher probability of adverse outcomes are ISS >25, GCS <8, head and neck and extremity injuries, lung contusions, abdominal injury, need for blood transfusions, and operative management. ED physicians should have a low threshold for escalating care in the presence of these factors. Serum PCT at admission has low utility as a prognostic factor in severely injured patients.
